# Association between pretreatment neutrophil‐to‐lymphocyte ratio and immune‐related adverse events due to immune checkpoint inhibitors in patients with non‐small cell lung cancer

**DOI:** 10.1111/1759-7714.14063

**Published:** 2021-06-26

**Authors:** Airi Fujimoto, Gouji Toyokawa, Yoshimichi Koutake, Shigeru Kimura, Yosei Kawamata, Kazuhisa Fukuishi, Koji Yamazaki, Sadanori Takeo

**Affiliations:** ^1^ Department of Pharmacy, Clinical Research Institute National Hospital Organization Kyushu Medical Center Fukuoka Japan; ^2^ Department of Thoracic Surgery, Clinical Research Institute National Hospital Organization Kyushu Medical Center Fukuoka Japan

**Keywords:** immune checkpoint inhibitor, immune‐related adverse event, neutrophil‐to‐lymphocyte rate, non‐small cell lung cancer

## Abstract

**Background:**

Immune checkpoint inhibitors (ICIs) have revolutionized the treatment of advanced or recurrent non‐small cell lung cancer (NSCLC). They cause immune‐related adverse events (irAEs), but the underlying mechanisms and predictors remain to be fully elucidated. In this retrospective study, we investigated the association between pretreatment neutrophil‐to‐lymphocyte ratio (NLR) and the occurrence of irAEs.

**Methods:**

The study involved 115 patients with NSCLC who started ICI‐only treatment in our hospital between January 2016 and April 2020.

**Results:**

Forty‐five patients (39.1%) had irAEs, and pretreatment NLR was significantly lower in the irAEs group than in the non‐irAEs group (2.8 vs. 4.1; *p* = 0.036). The cutoff value of the NLR was 2.86 (area under curve, 0.62; sensitivity, 0.56; specificity, 0.71), and the incidence rate of irAEs was significantly higher in the NLR < 2.86 group than in the NLR ≥2.86 group (*p* = 0.004; odds ratio [OR]: 3.12; 95% confidence interval [CI]: 1.43–6.84). The multivariate analysis showed that the NLR was significantly associated with the occurrence of irAEs (*p* = 0.016; OR: 2.69; 95% CI: 1.21–6.01).

**Conclusions:**

Low pretreatment NLR may be a predictive factor for the occurrence of irAEs. By focusing on the potential risk of irAEs in patients with a low pretreatment NLR, irAEs can be appropriately managed from an early period.

## Graphical abstract

Here, we report that low pretreatment NLR may be a predictive factor for the occurrence of irAEs in NSCLC patients. The cutoff value of the NLR was 2.86, and the univariate and multivariate analysis showed that the NLR was significantly associated with the occurrence of irAEs.

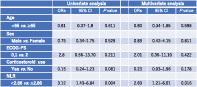

## INTRODUCTION

Immune checkpoint inhibitors (ICIs) have paved a new era for the treatment of advanced or recurrent non‐small cell lung cancer (NSCLC). Nivolumab, pembrolizumab, atezolizumab, durvalumab, and ipilimumab have been approved for the treatment of NSCLC, and monotherapy and combination therapy have been established as a standard course of care.^1^

ICIs exert antitumor effects by binding to inhibitory receptors expressed on T cells, or their ligands, and blocking the inhibitory signals of the immune system.^2^ An increase in immune functions induced by ICIs may cause immune‐related adverse events (irAEs) due to the disruption of immune function homeostasis.^3^ irAEs pose a risk of poor prognosis in severe cases, and ICI‐related mortality rate is in the range of 0.36%–1.23%.^4^ Appropriate treatment from an early period is required when patients experience irAEs; while there have been some reports on the predictive factors of irAEs,^5^, ^6^, ^7^, ^8^, ^9^ there are no established factors.

The neutrophil‐to‐lymphocyte ratio (NLR) is an index that reflects systemic inflammation. It can be easily calculated from the results of inexpensive routine blood tests; the prognostic value of NLR has been proven in cardiovascular diseases and infections.^10^, ^11^ Reportedly, the NLR can serve as a prognostic factor for solid tumors^12^, ^13^, ^14^ and a predictor of clinical benefits of ICIs.^15^, ^16^, ^17^ Furthermore, its relationship with irAEs has been investigated in recent years^8^, ^9^; however, there is still no unified view. In this study, we retrospectively investigated the association between NLR and the occurrence of irAEs in patients with advanced/recurrent NSCLC treated with ICIs.

## METHODS

### Patients

This retrospective study involved 115 patients with NSCLC who started treatment with ICIs between January 2016 and April 2020 in the National Hospital Organization Kyushu Medical Center. We excluded patients who had been treated with other ICIs before starting ICIs, patients who received ICIs in combination with chemotherapy, and patients whose peripheral blood neutrophils and lymphocytes were not measured within one week before the start of ICIs. We retrospectively surveyed the medical records of patients and investigated the irAEs. The NLR was calculated as follows: neutrophil count (/μl) / lymphocyte count (/μl). The Common Terminology Criteria for Adverse Events (CTCAE) version 5.0 was used to assess irAEs. The observation period was from January 2016 to September 2020.

### Ethical approval

This study was conducted in accordance with the ethical guidelines of the Declaration of Helsinki and the ethical guidelines for Medical and Health Research Involving Human Subjects. This study was approved by the investigational review board of the ethics committee of Kyushu Medical Center (approval no. 20C200).

### Dosage of ICIs

Nivolumab was administered every two weeks at a dose of 3 mg/kg until August 31, 2018, and 240 mg/bodyweight after November 1, 2018. During the two months between September 2018 and October 2018, a dose of either 3 mg/kg or 240 mg/bodyweight was chosen for each patient. Pembrolizumab was administered at a dose of 200 mg/bodyweight every three weeks, and atezolizumab was administered at 1200 mg/bodyweight every three weeks.

### Statistical analysis

In this study, patients were divided into two groups, namely, the irAEs and non‐irAEs groups. We used Fisher's exact test and the chi‐square test to compare the qualitative data between the groups, and Mann–Whitney U test to compare the quantitative data. Patients were dichotomized according to the cutoff values of NLR < 2.86 versus ≥2.86. The NLR cutoff value before the start of treatment was calculated using receiver operating characteristic (ROC) curve analysis. Multivariable logistic regression analysis was used to identify the predictive factors of irAEs.

Overall survival (OS) was defined as the period from the initiation of ICIs to patient death from any cause or to the last date of confirmation of survival based on the medical records. The OS was compared between the groups using the log‐rank test, and Holm method of the post‐hoc test was used to compare the OS among the four groups. Results with *p* < 0.05 were considered statistically significant. All statistical analyses were performed using EZR software version 1.53.^18^

## RESULTS

### Patient characteristics

This study involved 115 patients with advanced NSCLC, except for one patient with stage I who was inoperable because of poor lung function and was treated with an ICI after the failure of stereotactic irradiation. Forty‐five patients (39.1%) experienced irAEs (Table 1), with four patients experiencing multiple irAEs. There were no significant differences between the groups in terms of age, sex, Eastern Cooperative Oncology Group performance status (ECOG‐PS), smoking status, histopathological diagnosis, driver gene alteration, tumor proportion score, tumor stage, type of ICI, previous chemotherapy, previous thoracic radiotherapy, taking systemic corticosteroids, white blood cell count, neutrophil count, and lymphocyte count. The median (range) pretreatment NLR was 2.8 (0.9–12.0) and 4.1 (0.8–10.7) in the irAEs and non‐irAEs groups, respectively, and the difference was significant (*p* = 0.036).

**TABLE 1:** Patient characteristics of the non‐irAEs and irAEs groups

Factor		Non‐irAEs (*n* = 70) n (%)	irAEs (*n* = 45) n (%)	*p*‐value
Age (years)	Median (range)	69 (44–85)	68 (45–87)	0.866
Sex	Male	52 (74.3)	32 (71.1)	0.531
	Female	18 (25.7)	14 (31.1)	
ECOG‐PS	0	34 (48.6)	26 (57.8)	0.429
	1	28 (40.0)	17 (37.8)	
	2	8 (11.4)	2 (4.4)	
Smoking status	Never	16 (22.9)	16 (35.6)	0.200
	Ever	54 (77.1)	29 (64.4)	
Histological subtype	Adenocarcinoma	49 (70.0)	28 (62.2)	0.526
	Squamous cell carcinoma	18 (25.7)	13 (28.9)	
	Others	3 (4.3)	4 (8.9)	
*EGFR* mutation	Yes	9 (12.9)	7 (15.6)	0.918
	No	48 (68.6)	30 (66.7)	
	Unknown	13 (18.6)	8 (17.8)	
ALK rearrangement	Yes	1 (1.4)	1 (2.2)	0.612
	No	46 (65.7)	26 (57.8)	
	Unknown	23 (32.9)	18 (40.0)	
PD‐L1 (TPS)	< 1%	9 (12.9)	8 (17.8)	0.523
	1 ~ 49%	21 (30.0)	10 (22.2)	
	≥ 50%	17 (56.7)	8 (17.8)	
	Unknown	23 (76.7)	19 (42.2)	
Stage^a^	I	0 (0.0)	1 (1.9)	0.351
	II	0 (0.0)	0 (0.0)	
	III	4 (6.5)	6 (11.3)	
	IV	58 (93.5)	46 (86.8)	
ICIs	Nivolumab	38 (54.3)	27 (60.0)	0.703
	Pembrolizumab	16 (22.9)	11 (24.4)	
	Atezolizumab	16 (22.9)	7 (15.6)	
Number of previous chemotherapy	0	10 (14.3)	10 (22.2)	0.426
	1 or 2	47 (67.2)	25 (55.6)	
	≥ 3	13 (18.6)	10 (22.2)	
Previous thoracic radiotherapy	Yes	12 (17.1)	5 (11.1)	0.431
	No	58 (82.9)	40 (88.9)	
Corticosteroid use^b^	Yes	9 (12.9)	1 (2.2)	0.086
	No	61 (87.1)	44 (97.8)	
WBC (/μl)	Median (range)	6600 (2900–20 600)	6300 (2600–11 200)	0.281
Neut (/μl)	Median (range)	4397 (1676–17 222)	4315 (1027–8400)	0.139
Lym (/μl)	Median (range)	1162 (520–4870)	1280 (380–3254)	0.451
NLR	Median (range)	4.1 (0.8–10.7)	2.8 (0.9–12.0)	0.036

Abbreviations: ECOG‐PS, Eastern Cooperative Oncology Group Performance Status; ICIs, immune checkpoint inhibitors; irAEs, immune‐related adverse events; Lym, lymphocyte; NLR, neutrophil‐to‐lymphocyte rate; PD‐L1, programmed death‐ligand 1; TPS, tumor proportion score; WBC, white blood cell; Neut, neutrophil.

^a^ Tumor Nodes Metastasis Classification.

^b^ Administration of corticosteroids at the initiation of ICIs.

### Immune‐related adverse event

Table 2 shows the occurrence of irAEs and their severity. The most frequent irAEs was thyroid‐related events, followed by skin‐related events and interstitial lung diseases. Skin‐related events were rash and pruritus; none of the patients had vitiligo. The severity of thyroid‐related events was grade ≤ 2, and the treatment was interrupted in four patients, but treatment discontinuation was not required for any patient. The severity of interstitial lung disease was grade ≥ 3 in 5 patients, and treatment interruption and discontinuation were required for two and six patients, respectively. Other irAEs that required the discontinuation of treatment were colitis, encephalitis, hepatopathy, cardiac‐related events, thrombocytopenia, and hypoadrenocorticism. Colitis was considered to be immune‐related enteritis in all patients; all patients were treated with corticosteroids, except one patient, who was treated with infliximab. With respect to encephalitis, two patients presented decreased consciousness level of grade 3; with regard to cardiac‐related events, pericardial tamponade was observed in one patient and acute myocardial infarction in one patient. Two patients presented with immunogenic thrombocytopenia and one patient developed symptoms two months after ICI discontinuation.

**TABLE 2:** Summary of irAEs

irAEs	All grade n (%)	Grade, n (%)			ICI interruption n (%)	ICI discontinuation n (%)
		1	2	≥ 3		
Thyroiditis / hypothyroidism	16 (13.9)	4 (25.0)	12 (75.0)	‐	4 (25.0)	‐
Skin‐related events	14 (12.2)	9 (64.3)	5 (35.7)	‐	‐	‐
Interstitial lung disease	8 (7.0)	1 (12.5)	2 (25.0)	5 (62.5)	2 (25.0)	6 (75.0)
Colitis	5 (4.3)	1 (20.0)	2 (40.0)	2 (40.0)	‐	3 (40.0)
Encephalitis	2 (1.7)	‐	‐	2 (100)	‐	2 (100)
Cardiac‐related events	2 (1.7)	‐	‐	2 (100)	‐	2 (100)
Thrombocytopenia	2 (1.7)	‐	1 (50.0)	1(50.0)	‐	1 (50.0)
Hypoadrenocorticism	2 (1.7)	‐	‐	2 (100)	1 (50.0)	1 (50.0)
Hepatopathy	2 (1.7)	1 (50.0)	‐	1 (50.0)	‐	1 (50.0)
Renal dysfunction	1 (0.9)	1 (100)	‐	‐	1 (100)	‐

Abbreviations: ICI, immune checkpoint inhibitors; irAEs, immune‐related adverse events.

### Association between NLR and irAEs

**FIGURE 1:**
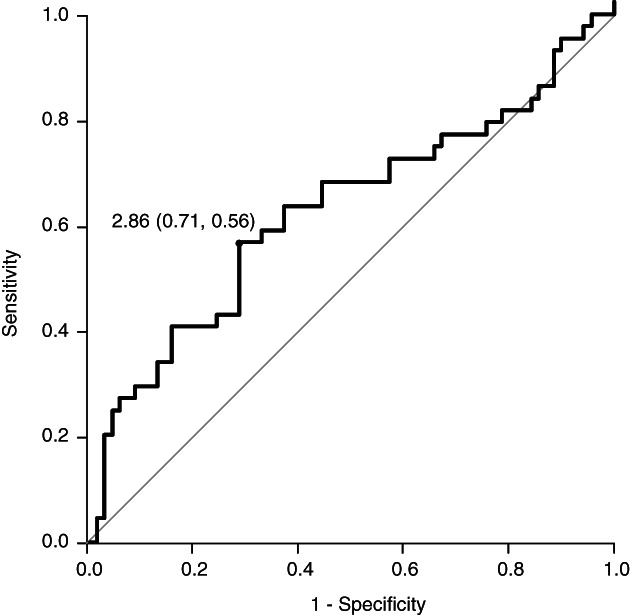
Receiver operating characteristic curve of pretreatment neutrophil‐to‐lymphocyte ratio (NLR) for the occurrence of immune‐related adverse events (irAEs). The cutoff value of pretreatment NLR for the occurrence of irAEs was 2.86 (area under curve, 0.62; 95% confidence interval [CI]: 0.50–0.73; sensitivity, 0.56; specificity, 0.71)

The cutoff value of pretreatment NLR for the occurrence of irAEs was 2.86 (area under curve, 0.62; 95% confidence interval [CI]: 0.50–0.73; sensitivity, 0.56; specificity, 0.71; Figure 1). Among 115 patients treated with ICIs, 70 (60.9%) had an NLR of ≥2.86, and 45 (39.1%) had an NLR of <2.86, and the univariate analysis showed that the occurrence rate of irAEs was significantly higher in the NLR < 2.86 group than in the NLR ≥2.86 group (*p* = 0.004; Table 3). The multivariate analysis revealed that the NLR < 2.86 can be an independent predictive factor for the occurrence of irAEs (*p* = 0.016; odds ratio [OR]: 2.69; 95% Cl: 1.21–6.01; Table 3). There was no significant difference between the grade of irAEs and level of NLR (grade 1, 2 vs. > 3; *p* = 0.577, date not shown).

**TABLE 3:** Univariate and multivariate logistic regression analyses of factors associated with irAEs

	Univariate analysis			Multivariate analysis		
	ORs	95% CI	*p*‐value	ORs	95% CI	*p‐*value
Age						
<65 vs. ≥65	0.81	0.37–1.8	0.611	0.796	0.34–1.85	0.596
Sex						
Male vs. female	0.75	0.34–1.75	0.529	0.889	0.42–2.15	0.811
ECOG‐PS						
0,1 vs. 2	2.80	0.56–13.70	0.211	2.01	0.36–11.10	0.422
Corticosteroid use^a^						
Yes vs. No	0.154	0.15–1.23	0.081	0.227	0.03–1.96	0.178
NLR						
<2.86 vs. ≥2.86	3.12	1.43–6.84	0.004	2.69	1.21–6.01	0.016

^a^ Administration of corticosteroids at the initiation of ICIs.

Abbreviations: 95% Cl, 95% confidence interval; ECOG‐PS, Eastern Cooperative Oncology Group Performance Status; irAEs, immune‐related adverse events; NLR, neutrophil‐to‐lymphocyte rate; ORs, odds ratios.

### Overall survival

**FIGURE 2:**
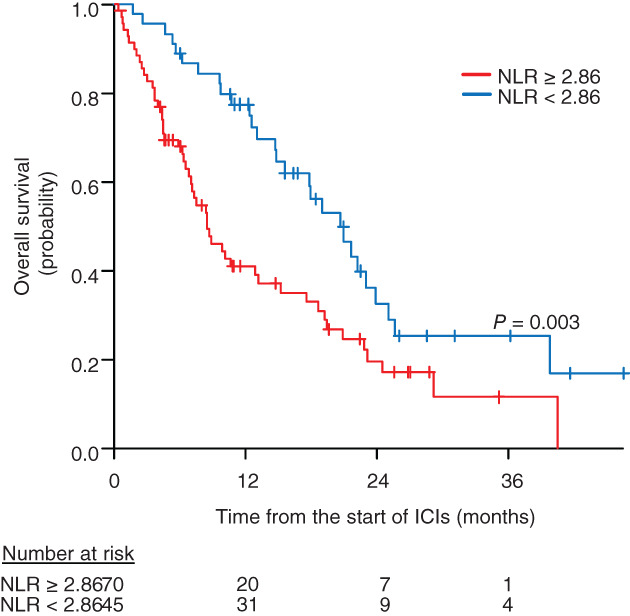
Overall survival (OS) according to pretreatment neutrophil‐to‐lymphocyte ratio (NLR). The median OS was 20.6 months (95% confidence interval [CI]: 14.8–23.9 months) in the NLR < 2.86 group and 8.3 months (95% CI: 6.3–12.7 months) in the NLR ≥2.86 group (*p* = 0.002)

**FIGURE 3:**
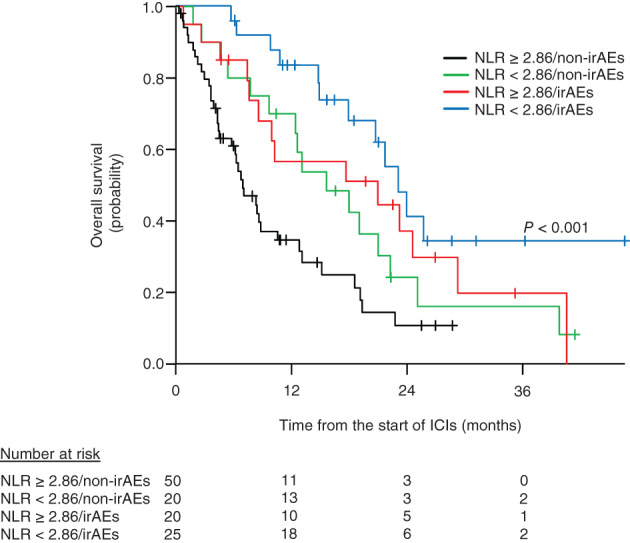
Overall survival (OS) according to pretreatment neutrophil‐to‐lymphocyte ratio (NLR) and immune‐related adverse events (irAEs). The median OS was 6.9 months (95% confidence interval [CI]: 4.5–10.5 months) for NLR ≥2.86/non‐irAEs, 15.5 months (95% CI: 7.6–22.1 months) for NLR < 2.86/non‐irAEs, 20.7 months (95% CI: 7.4–29.0 months) for NLR ≥2.86/irAEs, and 23.0 months (95% CI: 17.8 months to not available) for NLR < 2.86/irAEs. All, *p* < 0.001; NLR ≥2.86/non‐irAEs versus NLR < 2.86/irAEs, *p* < 0.001; NLR ≥2.86/non‐irAEs versus NLR < 2.86/non‐irAEs, *p* = 0.199; NLR ≥ 2.86/non‐irAEs versus NLR ≥ 2.86/irAEs, *p* = 0.082; NLR < 2.86/non‐irAEs versus NLR < 2.86/irAEs, *p* = 0.199; NLR < 2.86/non‐irAEs versus NLR ≥2.86/irAEs, *p* = 0.679; NLR ≥2.86/irAEs versus NLR < 2.86/irAEs, *p* = 0.199

The median OS was 20.6 (95% CI: 14.8–23.9) months in the NLR < 2.86 group and 8.3 (95% CI: 6.7–13.0) months in the NLR ≥2.86 group (*p* = 0.003; Figure 2). Among 70 patients with a pretreatment NLR of ≥2.86, 20 (28.6%) developed irAEs (NLR ≥2.86/irAEs), and 50 (71.4%) patients did not experience irAEs (NLR ≥2.86/non‐irAEs). Among 45 patients with a pretreatment NLR of <2.86, 25 (55.6%) developed irAEs (NLR < 2.86/irAEs), and 20 (44.4%) patients did not experience irAEs (NLR < 2.86/non‐irAEs). The median OS was 20.5 (95% CI: 7.4–29.0) months for NLR ≥2.86/irAEs, 6.9 (95% CI: 4.5–10.5) months for NLR ≥2.86/non‐irAEs, 23.0 months (95% CI: 17.8 months to not available) for NLR < 2.86/irAEs, and 15.5 (95% CI: 7.7–22.1) months for NLR < 2.86/non‐irAEs (*p* < 0.001; Figure 3).

### Discussion

In this study, pretreatment NLR was significantly lower in the irAEs group than in the non‐irAEs group, with a cutoff value of 2.86. Additionally, the occurrence rate of irAEs was significantly higher in the NLR < 2.86 group than in the NLR ≥2.86 group. This result is in agreement with the results of Pavan et al.^8^ and Eun et al.^9^ who showed that a pretreatment NLR of <3 is a risk factor for the occurrence of irAEs. In these studies, the NLR cutoff value was set to 3. This value was not calculated as a risk factor for the development of irAEs, but was considered from previous studies reporting the association between NLR and prognosis of solid tumors and ICI treatment outcomes.^19^, ^20^, ^21^, ^22^, ^23^ We focused on irAEs and calculated the cutoff value of NLR, and the value was close to 3. Pavan et al.^8^ reported the findings of a study in Europe and Eun et al.^9^ investigated multiple cancer types. It has been reported that the types of irAEs and their occurrence rates differ depending on race and cancer type.^24^, ^25^ Our findings demonstrated the association between low pretreatment NLR and high occurrence rates of irAEs in Japanese patients with NSCLC. As far as we know, this is the first study to have demonstrated the significance of NLR in predicting irAEs in Japanese patients with NSCLC. On the basis of these results, we speculate that the NLR may be an important factor for predicting irAEs, regardless of race and cancer type.

We showed that OS of patients with a pretreatment NLR of <2.86 was significantly longer than that of patients with a pretreatment NLR of ≥2.86. This indicates that patients with a pretreatment NRL of <2.86 are at a high risk of developing irAEs, but good clinical outcomes can be expected. In addition, the OS of patients with NLR < 2.86/irAEs was significantly prolonged compared with that of patients with NLR ≥2.86/non‐irAEs. Although no significant difference was observed, the survival curve suggested that the OS of patients in the NLR < 2.86/irAEs group may be longer than that of patients in the NLR < 2.86/non‐irAEs group. This difference may be because a lower NLR has been associated with a better outcome of ICI treatment,^16^, ^17^ and the development of irAEs has been found to be related to survival benefits in patients with NSCLC.^26^, ^27^, ^28^

Lymphocytes, mainly T cells, are involved in the antitumor immune response and suppress the growth of tumor cells.^29^ In contrast, neutrophils promote tumor development by acting on tumor cells and the tumor microenvironment,^30^ and peripheral neutrophil counts have been reported to correlate with intertumoral neutrophil counts.^31^ Furthermore, the increase in peripheral neutrophils adversely affects the cytotoxic activity of lymphocytes and may attenuate the antitumor response.^30^ The main mechanism of the occurrence of irAEs is considered to be the damage to self‐tissues due to the activation of lymphocytes that react with self‐antigens following the administration of ICIs.^32^ Low NLRs reflect low peripheral neutrophil counts and high lymphocyte counts, indicating the maintenance of antitumor response of the immune system as well as the increased risk of the presence of lymphocytes that inappropriately react to self‐antigens. Therefore, we consider that a low pretreatment NLR leads to the improvement of positive outcomes of ICI treatment and the increased risk of irAEs.

Biomarkers for irAEs have been extensively investigated. To date, such biomarkers including HLA genotypes, soluble serum proteins, and gut microbiota have been reported to predict irAEs.^33^, ^34^, ^35^ However, evaluation of these biomarkers is associated with clinical challenges, such as accessibility, complexity, and high cost. Compared with these biomarkers, NLR is easy to measure and inexpensive. The NLR could be a clinically cost‐effective and useful predictive factor for irAEs.

There were some limitations to the study, including its retrospective nature, single‐institutional design, and limited sample size. Large‐scale, multi‐institutional, retrospective, and prospective studies on the relationship between the NLR and the occurrence of irAEs are warranted. Additionally, we cannot deny that the association between a low NLR and higher incidence of irAEs may be caused by the longer OS in a low NLR group; longer OS usually means exposure to more doses of ICIs. The relationship between the duration of exposure or the cumulative dose of ICIs and irAEs has not been clarified, and this should be investigated in the future.

In conclusion, a low NLR may be a predictive factor for the onset of irAEs. The NLR can be measured in an inexpensive and easy manner through routine clinical practice. By focusing on the potential risk of irAEs in patients with a low pretreatment NLR, irAE management can be appropriately managed from an early period. We consider that this could maximize the benefits of ICI treatment.

## CONFLICT OF INTEREST

All authors declare no potential conflict of interest.
